# Drug–drug Interaction between Pravastatin and Gemfibrozil (Antihyperlipidemic) with Gliclazide (Antidiabetic) in Rats

**DOI:** 10.4103/0975-1483.63157

**Published:** 2010

**Authors:** CM Sultanpur, S Satyanarayana, NS Reddy, KE Kumar, S Kumar

**Affiliations:** *Pharmacology Division, Government College of Pharmacy, Bangalore - 560 027, India*; 1 *Pharmacology Division, University College of Pharmaceutical Sciences, Andhra University, Visakhapatnam - 530 003, India*

**Keywords:** Diabetes, drug–drug gliclazide, gemfibrozil, interaction, pravastatin, pharmacodynamics

## Abstract

Diabetes mellitus is a condition of increased blood glucose level in the body. Antihyperlipidemic drugs like statins and fibrates are widely used for prophylactic treatment in dyslipideamia and atherosclerosis. Diabetic dislipidemia exists with increased triglycerides, low HDL and high LDL levels. Hence, with oral hypoglycemic drugs, the addition of a lipid-lowering drug is necessary for controlling dislipidemia. In such a situation, there may be chances of drug–drug interactions between antidiabetic and antihyperlipidemic drugs. The present study is planned to evaluate the safety of gliclazide (antidiabetic) in the presence of pravastatin and gemfibrozil (antihyperlpidemic) in rats. Studies in normal and alloxan-induced diabetic rats were conducted with oral doses of gliclazide and their combination with pravastatin and gemfibrozil, with an adequate washout period in between the treatments. Blood samples were collected in rats by retroorbital puncture at 0, 1, 2, 3, 4, 6, 8, 10 and 12 h. All the blood samples were analyzed for glucose by GOD –POD. Gliclazide (½ TD) produced hypoglycemic activity in normal and diabetic rats, with peak activity at 2 and 8 h. Pravastatin (TD) + gemfibrozil (TD) combination treatment increased the hypoglycemic effect of gliclazide in normal rats or diabetic rats when administered together. The interaction observed due to inhibition of both the enzymes (CYP 450 2C9 and CYP 450 3A4) responsible for the metabolism of gliclazide showed increased half-life, which was seen in the present study. Because concomitant administration of gliclazide with provastatin and gemfibrozil in diabetes is associated with atherosclerosis, it should be contraindicated or used with caution.

## INTRODUCTION

A study of the mechanisms of drug interactions is of much value in selecting the drug combinations to provide rational therapy. The drug interaction studies assume much importance, especially for drugs that have a narrow margin of safety and where the drugs are used for a prolonged period of time. Diabetes mellitus is one such metabolic disorder that needs treatment for prolonged periods, and maintenance of normal blood glucose level is very important in this condition because both hyperglycaemia and hypoglycaemia are unwanted phenomena.

Sulfonylureas are the drugs of choice in the treatment of type 2 diabetes.[1] Currently, gliclazide, a second-generation sulfonylurea, is preferred in therapy because of its selective inhibitory activity toward pancreatic K^+^ ATP channels,[2] low incidence of producing severe hypoglycemia[3] and other hemobiological effects.[4] It is well established that sulfonylureas produce insulin secretion and improve tissue utilization of glucose at the cellular level,[5] which was responsible for lowering of the blood glucose level. The sulfonylureas and related drugs used in type 2 diabetes stimulate insulin by closing K^+^ ATP channels in pancreatic β cells.

Antihyperlipidemic drugs like statins and fibrates are widely used for prophylactic treatment in dyslipidemia and atherosclerosis. Pravastatin is known to inhibit liver microsomal enzyme CYP 450 3A4,CYP 2C9 and CYP 2D6.[6,7] Gemfibrozil is metabolized by the hepatic cytochrome CYP 450 2C9.[8]

Hence, there is a higher possibility of pravastatin and gemfibrozil for inhibition of the metabolism of gliclazide, because they are also metabolized by both CYP 450 2C9 and CYP 3A4.[9]

Because concomitant administration of gliclazide with pravastatin and gemfibrozil in diabetes is associated with atherosclerosis, there is every possibility for drug–drug interaction with enhanced or decreased gliclazide activity, which is unwanted.

The safety of the above drug combinations with respect to blood glucose is not known and needs to be established by preclinical and clinical studies. This study is planned to establish the safety of the drug combinations in the rat model with respect to blood glucose level and find out the mechanisms responsible for the interaction, if any.

## MATERIALS AND METHODS

### Drugs and chemicals

Gliclazide, gemfibrozil and pravastatin are gift samples from Micro Labs (Bangalore, India) and Biocon (Bangalore, India). Alloxan monohydrate was purchased from Sigma Aldrich (Bommasandra, Jigni, and Bangalore, India). Glucose kits of span diagnostics were procured from local suppliers. All of the chemicals used are of analytical grade.

### Animals

Albino rats of either sex, weighing between 160 and 280 g, procured from Drugs Testing Lab, Bangalore, India, were used in the study. They were maintained under standard laboratory conditions at an ambient temperature of 25 ± 2°C, with 12-h light/12-h dark cycles. They were fed with standard pellet diet (Venkateshwar Enterprises Pvt. Ltd., Bangalore, India) and water *ad libitum*. Animals were fasted for 18 h before the experiment and, during the experiment, they were withdrawn from food and water. Prior approval for conducting experiments on rats was obtained from our Institutional Animal Ethics Committee and our laboratory is approved by CPCSEA, Govt. of India (Regd. No. GCP/CPCSEA/04/2005-06).

### Method

#### Pharmacodynamic study in normal or diabetic rats

A group of six rats were administered (½ TD) 0.72 mg/200 g of bd wt of gliclazide orally. The same group was administered with pravastatin (TD) 0.72 mg/200 g bd wt and gemfibrozil (TD) 21.6 mg/200 g bd wt orally in combination with gliclazide. A 1-week washout period was maintained between the treatments. The same treatment was repeated in a group of six alloxan-induced diabetic rats. Blood samples were withdrawn by retroorbital puncture[10] at 0, 1, 2, 3, 4, 6, 8, 10 and 12 h and were analysed for blood glucose by the GOD or POD method[11] using commercial glucose kits (Span Diagnostics).

#### Induction of diabetes

Diabetes was induced in rats by the administration of alloxan monohydrate in two doses, i.e. 100 mg and 50 mg/kg bd wt intraperitoneally for two consecutive days.[12]

#### Data and statistical analysis

Data were expressed as mean ± standard error of mean (SEM). The significance was determined by applying Student’s paired *t*-test.

## RESULTS

**Figure 1:**
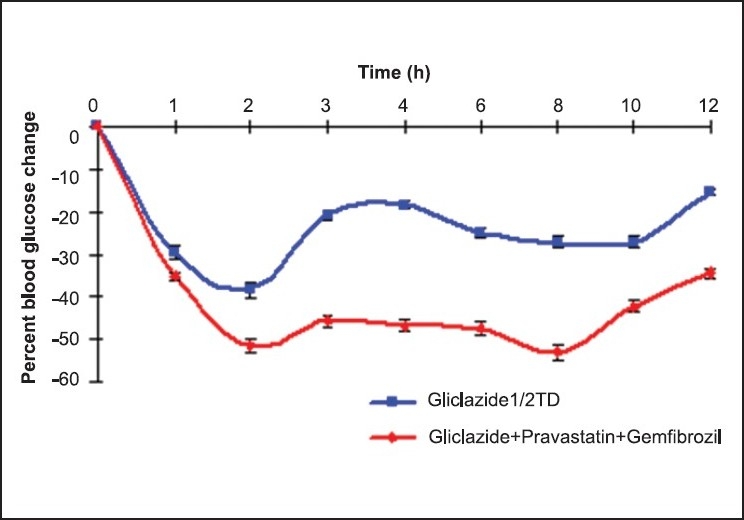
The mean percent blood glucose change with gliclazide alone and gliclazide + pravastatin + gemfibrozil combination in normal rats (n = 6)

**Figure 2:**
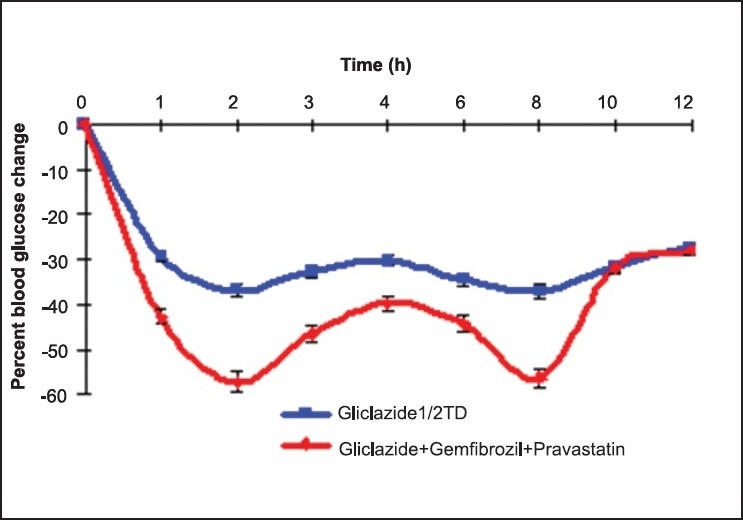
The mean percent blood glucose change with gliclazide alone and gliclazide + pravastatin + gemfibrozil combination in diabetic rats (n = 6)

Gliclazide produced biphasic hypoglycaemic activity, with maximum reduction of 38.51 ± 1.65% and 37.29 ± 3.48% at 2 and 8 h in normal rats [Table 1, Figure 1], and hypoglycemic activity with maximum reduction of 37.61 ± 0.38% and 38.12 ± 1.47% at 2 and 8 h in diabetic rats [Table 2, Figure 2], respectively. Gliclazide, when given in combination with pravastatin and gemfibrozil, produced increased hypoglycemic effect with maximum reduction of 51.44 ± 2.10% and 53.19 ± 1.72% and 57.18 ± 0.81% and 56.27 ± 0.56% in the blood glucose in normal and diabetic rats at 2 and 8 h, respectively.

**Table 1:** Mean percent blood glucose change after oral administration of gliclazide alone and gliclazide + pravastatin + gemfibrozil combination in normal rats (n = 6)

Time (h)	Gliclazide (½ TD)	Gliclazide (½ TD) + Pravastatin (TD) + Gemfibrozil (TD)
0	-	-
1	-29.89 ± 2.24	-35.33 ± 1.90
2	-38.51 ± 1.65	-51.44 ± 2.10**
3	-21.02 ± 1.65	-45.73 ± 1.65***
4	-18.50 ± 3.40	-46.67 ± 1.63***
6	-25.12 ± 1.79	-47.68 ± 2.53***
8	-37.29 ± 3.48	-53.19 ± 1.72***
10	-27.45 ± 3.48	-42.41 ± 3.24*
12	-15.54 ± 1.22	-34.52 ± 2.56*

*** Significant at *P*<0.001

** Significant at *P*<0.01

* Significant at *P*<0.05 compared to gliclazide control

**Table 2:** Mean percent blood glucose change after oral administration of gliclazide alone and gliclazide + pravastatin + gemfibrozil combination in diabetic rats (n = 6)

Time (h)	Gliclazide (½ TD)	Gliclazide (½ TD) + Pravastatin (TD) + Gemfibrozil (TD)
0	-	-
1	-36.94 * 1.11	-42.70 + 0.72**
2	-37.61 * 0.38	-57.18 + 0.81***
3	-31.35 * 1.85	-46.45 * 0.60***
4	-26.47 * 1.96	-39.67 + 0.83***
6	-36.12 * 1.23	-44.32 + 0.68**
8	-38.12 * 1.47	-56.27 + 0.56***
10	-32.28 * 1.52	-31.70 + 0.86
12	-25.48 * 2.64	-28.02 + 0.70

*** Significant at *P*<0.001

** Significant at *P*<0.01

*Significant at *P*<0.05 compared to gliclazide control

## DISCUSSION

Drug interactions are usually seen in clinical practice, and the mechanisms of interactions are usually evaluated in animal models. We studied the influence of gemfibrozil and pravastatin on the pharmacodynamics of gliclazide in normal and diabetic rats. The normal rat model served to quickly identify the interaction and the diabetic rat model served to validate the same response in the actually used condition of the drug.

Gliclazide produced a biphasic response in the rat model when administered alone, which may due to its biliary excretion and enterohepatic circulation in rats[13,14] and in humans.[15] Gliclazide is known to produce a hypoglycemic activity by pancreatic[16] (stimulating insulin secretion by blocking K^+^channels in the pancreatic β cells) and extrapancreatic[17] (increasing tissue uptake of glucose) mechanisms.

Pravastatin and gemfibrozil enhanced hypoglycemic effects produced by gliclazide in normal and diabetic rats when administered in combination. This may be due to their activity on insulin secretion.

Because pravastatin is known to inhibit liver microsomal enzymes CYP 450 3A4, CYP 450 2C9 and CYP 450 2D6 and gemfibrozil is known to be metabolized to a major extent by CYP 450 2C9, by which gliclazide is also metabolized primarily and, to a lesser extent, by CYP 450 3A4, the interaction might be at the level of their metabolism. Pravastatin and gemfibrozil might compete with gliclazide for metabolism by CYP 450 2C9 and CYP 450 3A4 and delay the metabolism of gliclazide, leading to its enhanced effect. The metabolites of gliclazide, namely hydroxy and carboxy gliclazide, are pharmacologically inactive. Hence, inhibition of gliclazide metabolism improves its unchanged level and pharmacological action, which is seen in the present study.

However, the drug pravastatin and gemfibrozil combination treatment did not change the pattern of biphasic response of gliclazide, indicating that it did not interfere with the reabsorption of gliclazide in its enterohepatic circulation in rats.

The drug profile of gemfibrozil shows that it is a highly protein-bound drug, and about 98.6% or greater (Hamberger)[18] was bound to plasma proteins. Further, gliclazide was reported to interact with highly protein-bound drugs.[19] Because both gliclazide and gemfibrozil are highly protein-bound drugs, gemfibrozil might displace other drugs from protein-binding sites, leading to its enhanced response. In the presence of the above drugs, sustained hypoglycemic activity of gliclazide was observed compared to gliclazide control.

## CONCLUSION

The interaction was observed in normal and diabetic rats. It is likely to occur in humans also. Hence, the combination gliclazide (½ TD) + pravastatin (TD) + gemfibrozil (TD) should be contraindicated or used with caution in a clinical situation.
